# Retting and degumming of natural fibers by pectinolytic enzymes produced from *Bacillus tequilensis* SV11-UV37 using solid state fermentation

**DOI:** 10.1186/s40064-016-2173-x

**Published:** 2016-05-04

**Authors:** Swarupa Rani Chiliveri, Sravanthi Koti, Venkateswar Rao Linga

**Affiliations:** Department of Microbiology, Osmania University, Hyderabad, Telangana State 500007 India

**Keywords:** Pectate lyase, Polygalacturonase, Retting, Degumming, Solid state fermentation

## Abstract

The present study demonstrated the simultaneous production and optimization of pectinolytic enzymes (pectate lyase and polygalacturonase) under SSF from *Bacillus tequilensis* SV11-UV37 using wheat bran as a substrate, which is commercially viable and cost-effective. Optimization by one variable-at-a-time-approach showed a maximum yield of pectate lyase (1371.25 U/gds) and polygalacturonase (85.45 U/gds) with wheat bran using 80 % (v/w) moisture, 0.7 mm particle size, 20 % (v/w) inoculum, 1 % (w/w) pectin at 37 °C, pH 6 and 72 h of incubation. In addition, optimization using central composite design achieved 1.6-fold improvement in both pectate lyase (1828.13 U/gds) and polygalacturonase (105.55 U/gds) yield at optimum levels of pectin (3 %, w/w), inoculum size (20 %, v/w) and moisture level (80 %, v/w). Further, Retting studies concluded that the enzyme mixture was efficient in separating the whole fiber from kenaf and part (>75 %) from sunn hemp. In degumming of sunn hemp fibers, amount of galacturonic acid released and percentage weight loss was higher in successive alkali and enzymatic treatment than their independent treatments. The scanning electron microscopic analysis also confirmed that alkali followed by enzymatic treatment effectively removed non-cellulosic gummy material from the fiber; hence, this enzyme mixture may find feasible applications in the fiber and textile industry.

## Background

Pectic substances are major constituents of the middle lamellae of plant cells that are present in the form of calcium and magnesium pectate, and they are high molecular weight, negatively charged and acidic polysaccharides (Jayani et al. 2005; Murad and Azzaz 2011). Pectins are polymers of d-galacturonic acid residues joined by α-1,4 glycosidic linkages, with a few number of xylose, arabinose, galactose and l-rhamnose molecules as their side chains (Gummadi and Kumar 2006). The enzymes that hydrolyze pectic substances are broadly classified as pectinases or pectinolytic enzymes. These include two main groups: methyl esterases, which catalyze the removal of the methoxyl group from pectin to form pectate, and depolymerases (hydrolases and lyases), which split the backbone of both pectin and pectate by hydrolytic cleavage (polymethyl galacturonases and polygalacturonases) or trans-eliminative cleavage (pectin lyases and pectate lyases) (Yuan et al. 2011).

Two fermentation methods, solid state fermentation (SSF) and submerged fermentation (SmF), have been used to produce pectinases. The aim of SSF is to achieve the highest substrate concentration for fermentation by bringing a cultivated fungus or bacterium into close contact with the insoluble substrate. Due to the lower cost of the method, SSF has been favored over SmF for these enzymes production. One great advantage of SSF has always been the possibility of using a substrate that is abundant and cheap, which is not the case in SmF. Many studies have employed agricultural residues as substrates (Tivkaa et al. 2013; Meenakshisundaram 2012). Among such agricultural residues, wheat bran is the most prominent since it is produced worldwide in huge quantities as an important by-product of the cereal industry and has been one of the most common substrates employed in biotechnological processes (Kashyap et al. 2003; Martins et al. 2002).

“One-factor-at-a-time” approach is a traditional method that has been used to optimize process variables, which is time-consuming, effortful and doesn’t reveal the interactions between the variables (Gummadi and Kumar 2006). An alternative and more effective approach is the response surface methodology (RSM) (Gonçalves et al. 2012). This approach includes factorial experimental design and regression analysis, which help evaluate the effective factors and their interactions and determine the optimum values of variables for the desired response (Zambare 2011).

Alkaline pectinases have attracted textile industry due to their ample applications in the retting and degumming of plant fibers such as buel, flax, ramie, sunn hemp, and jute (Saleem et al. 2008). Retting is a fermentation process, where certain bacteria (e.g., *Clostridium*, *Bacillus*) and fungi (e.g., *Aspergillus*, *Penicillium*) release the fiber by decomposing pectin present in the bark. Degumming is a method, which removes heavily coated, non-cellulosic gummy material from the cellulose part of the plant fibers. The fibers contain gum that should be removed before its use for textile manufacture. A high pH optimum of pectinases from microorganisms is advantageous for degumming of plant fibers since it prevents contamination and additionally permits adopting an open fermentation system (Hoondal et al. 2002). Hence, this study focused on the production and optimization of alkaline pectate lyase (polygalacturonate lyase-PGL) and polygalacturonase (PG) using *Bacillus tequilensis* SV11-UV37 along with the application in retting and degumming of natural fibers for their potential use in the fabric or textile industry.

## Methods

### Microorganism

A UV mutant (UV37) of pectinolytic microorganism *B. tequilensis* SV11 (GenBank accession no. JX473585 and culture deposition no. MTCC-11716) was used for the current study, which we have previously isolated and identified in our research laboratory (Chiliveri et al. 2012).

### Solid state fermentation for PGL and PG production by *Bacillus tequilensis* SV11-UV37

SSF was performed in 250 mL Erlenmeyer flasks with 10 g of each substrate moistened with distilled water, autoclaved at 15 lbs pressure and inoculated with 2 mL of 18 h nutrient broth culture. Selection of substrate for the PGL and PG production by *B. tequilensis* SV11-UV37 was done by using various agro-residues, viz., rice bran, cotton seed cake, corn cob, coconut cake, ground nut cake and wheat bran with different moisture levels (50–80 % v/w) by incubating up to 96 h at 37 °C.

### Optimization of process parameters by one-variable-at-a-time approach

Various process parameters for the PGL and PG production using wheat bran under SSF were optimized by varying one factor at a time and keeping other variables constant. The parameters optimized were particle size of the substrate (<0.5, 0.7, 1.0 and 2.0 mm), temperature (30, 35, 37, 40 and 45 °C), pH of the moistening solution (6.0, 6.5, 7.0, 7.5 and 8.0), inoculum size (10, 20, 30, 40 and 50 % v/w) and nutritional parameters such as 1 % w/w of different carbon sources [glucose, galacturonic acid (GA), sucrose, starch, pectin, polygalacturonic acid (PGA), xylose, arabinose and lactose], 1 % w/w of various organic and inorganic nitrogen sources [yeast extract, peptone, urea, tryptone, (NH_4_)_2_HPO_4_, NH_4_NO_3_, NH_4_Cl and (NH_4_)_2_SO_4_] and 0.05 % w/w of different metal ions (CaCl_2_, MnSO_4_, MgSO_4_, CoCl_2_, FeCl_3_, ZnSO_4_, CuSO_4_, KNO_3_ and NaCl).

### Optimization of crucial parameters by response surface methodology (central composite design)

RSM is advantageous over conventional optimization techniques since it reduces the number of experiments and time required. It has been widely used for optimizing process variables for pectinase production (Swain et al. 2009; Songpim et al. 2010). In this study, the influence of pectin, inoculum size and moisture level on PGL and PG production was evaluated using a central composite design (CCD) under SSF. The levels of all the three parameters were fixed based on the results of the single parameter optimization (one-variable-at-a-time approach). CCDs are response surface designs that can fit a full quadratic model. The influence of each variable on enzyme production was tested at five different levels viz., −α, −1, 0, +1 and +α. A group of 20 experiments (Table 1) was carried out in triplicates and the mean value was taken for statistical analysis. The data obtained from RSM were subjected to the analysis of variance (ANOVA). The results obtained were substituted in the following second order polynomial equation that represents the behavior of the system.

$$\begin{aligned}{\text{Y}}& = \upbeta_{0} + \upbeta_{1} {\text{A}} + \upbeta_{2} {\text{B}} + \upbeta_{3} {\text{C}} + \upbeta_{1} \upbeta_{1} {\text{A}}^{2} + \upbeta_{2} \upbeta_{2} {\text{B}}^{2} \\&\quad + \upbeta_{3} \upbeta_{3} {\text{C}}^{2} + \upbeta_{1} \upbeta_{2} {\text{AB}} + \upbeta_{1} \upbeta_{3} {\text{AC}} + \upbeta_{2} \upbeta_{3} {\text{BC}} \end{aligned}$$

where Y is the response variable, β_0_ is the intercept, β_1_, β_2_, β_3_ are linear coefficients, β_1,1_, β_2,2_, β_3,3_ are squared coefficients, β_1,2_, β_1,3_, β_2,3_ are interaction coefficients and A, B, C, A^2^, B^2^, C^2^, AB, AC, BC are level of independent variables. The data analysis and response surface graph generation were done using the statistical software, Design expert (version 8.0.5, Stat-Ease Inc. Minneapolis, USA).

**Table 1:** Central composite design with observed and predicted responses of PGL and PG production under SSF by *Bacillus tequilensis* SV11-UV37

Run	A: Pectin (%)	B: Inoculum size (v/w)	C: Moisture level (%)	PGL observed (U/gds) ± SD	PGL Predicted (U/gds)	PG observed (U/gds) ± SD	PG Predicted (U/gds)
1	2.00	40.00	60.00	1401.25 ± 11.5	1370.02	91.97 ± 1.6	90.12
2	0.32	40.00	60.00	1534.38 ± 17.0	1562.74	97.365 ± 2.5	96.95
3	1.00	20.00	40.00	900.875 ± 5.2	867.29	91.395 ± 1.3	89.07
4	3.00	60.00	40.00	1448.75 ± 7.75	1427.84	72.15 ± 1.6	70.01
5	2.00	40.00	60.00	1341.88 ± 5.0	1370.02	88.4 ± 0.9	90.12
6	1.00	60.00	40.00	1802.5 ± 18.5	1786.04	95.03 ± 1.4	96.34
7	2.00	6.36	60.00	1293.75 ± 6.0	1334.63	85.43 ± 1.6	87.36
8	2.00	40.00	93.64	1220 ± 5.75	1234.40	102.1 ± 1.7	101.56
9	3.68	40.00	60.00	1533.13 ± 8.0	1558.93	81.03 ± 1.7	82.74
10	2.00	73.64	60.00	1693.13 ± 18.0	1706.42	80.79 ± 1.5	80.15
11	2.00	40.00	26.36	785 ± 5.5	824.77	80.96 ± 1.4	82.79
12	3.00	20.00	40.00	940 ± 8.25	913.02	76.93 ± 1.5	76.26
13	2.00	40.00	60.00	1411.88 ± 11.0	1370.02	91.19 ± 1.0	90.12
14	2.00	40.00	60.00	1343.75 ± 19.5	1370.02	88.31 ± 1.2	90.12
15	3.00	60.00	80.00	1334.38 ± 4.0	1329.66	86.07 ± 1.2	87.48
16	2.00	40.00	60.00	1378.75 ± 19.0	1370.02	91.4 ± 1.9	90.12
17	1.00	60.00	80.00	1391.25 ± 11.5	1379.93	91.83 ± 0.6	91.58
18	3.00	20.00	80.00	1828.13 ± 20.0	1806.28	105.55 ± 2.0	103.33
19	1.00	20.00	80.00	1470 ± 25.0	1452.61	92.69 ± 3.0	93.91
20	2.00	40.00	60.00	1351.88 ± 10.0	1370.02	89.67 ± 1.0	90.12

*SD* standard deviation

### Enzyme extraction

The enzyme was extracted from fermented wheat bran by adding distilled water (1:10) and incubating at 150 rpm for 1 h in a rotary shaker. The homogenate was filtered and centrifuged at 10,000 rpm for 15 min at 4 °C. The supernatant obtained was treated as an enzyme source.

### Enzyme assays

#### Pectate lyase assay

Pectate lyase activity was determined spectrophotometrically by measuring the increase in absorbance at 235 nm (Songpim et al. 2010). The reaction mixture (1 mL) containing 25 mM Tris–HCl buffer and 1 mM CaCl_2_ of pH 9.0, 0.4 % (w/v) PGA and 0.04 mL of crude enzyme was incubated at 60 °C for 10 min. The reaction was stopped by adding 4 mL of 0.01 M HCl to the reaction mixture. Crude enzyme was inactivated by boiling for 10 min to use as a control in the reaction. One unit of pectate lyase corresponds to the amount of enzyme which lyses 0.4 % PGA solution and releases products with an absorbance increase of 0.2 at 235 nm within 10 min under the standard assay conditions.

#### Polygalacturonase assay

The reaction mixture containing 0.1 mL of 0.4 % PGA (Sigma Aldrich Pvt. Ltd) and 0.1 mL of supernatant was incubated at 60 °C (pH 9.0) for 10 min. Polygalacturonase activity was determined by measuring the amount of reducing groups released using the DNS method (Miller 1959). One unit of enzyme activity was defined as 1 µmol of GA released per minute under the standard assay conditions.

### Application studies

#### Retting of kenaf and sunn hemp fibers

Retting of natural fibers from mature sticks of kenaf (*Hibiscus cannabinus*) and sunn hemp (*Crotalaria juncea*) was studied by using a reported method (Yadav et al. 2009). An about 10 cm long stick (stem material) of each natural fiber was entirely submerged in test tubes containing 10 mL of 25 mM Tris buffer (pH 9.0). One test tube was made control labeled as C and contained 500 U of the deactivated enzyme for the two fibers. The enzyme was deactivated by heating it in a water bath at 100 °C for 15 min. The other test tubes were labeled for each fiber individually as E1, E2, E3, E4 and E5, which contains 100, 200, 300, 400 and 500 U of the enzyme respectively, and incubated at 37 °C for 12, 24 and 36 h under static conditions. After incubation, the sticks were shaken vigorously each with 10 mL hot water for 1 min. Then hot water was poured off and the resulting sticks were photographed. A modified version of the Fried test was used to check the fiber separation (Zhang et al. 2003), wherein the samples were visually graded on a scale from 0 to 6 (0—no fibers were released, 1—0 to 10 mm, 2—10 to 25 mm, 3—25 to 50 mm, 4—50 to 75 mm, 5—>75 mm and 6—all fibers were released from the 10 cm long straw). The average score of each incubation was considered as the degree of fiber separation.

#### Degumming of sunn hemp fibers

Dried and decorticated sunn hemp fiber was washed and boiled in water for 15 min before the chemical and enzymatic treatment (Kapoor et al. 2001).

##### Effect of temperature, enzyme dosage and reaction time on fiber treatment

To determine the optimum temperature for degumming, the fiber treatment was carried out at various temperatures ranging from 30 to 60 °C and the samples were collected at different time intervals (6, 12, 18 and 24 h) for the estimation of GA released. The enzyme dosage and reaction time for fiber treatment were optimized by treating the fiber with different doses of enzyme ranging between 100 and 500 U of the reaction mixture for 12 h at 50 °C. Chemical (alkali) treatment of fiber was performed by incubating 2 g sunn hemp fiber with 100 mL of 2 % (w/v) NaOH solution at 90 °C for 12 h in 250 mL conical flasks. A “successive chemical and enzymatic treatment” was conducted by using prior alkali treated sunn hemp bast fiber with optimized enzyme dose (300 U) and incubating at 50 °C for 12 h. The µmol of reducing equivalents of GA released and percent reduction in fiber weight were taken into consideration in assessing the amount of degumming.

##### Scanning electron microscopy

Small pieces of fixed sunn hemp fibers were placed on stubs mounted with silver tape and were sputter coated with gold using HITACHI E-1010 ion sputter at 30 kV. The gold coated stubs were examined under a scanning electron microscope (HITACHI S-3700 N) at 15 kV at various magnifications.

## Results and discussion

### Solid state fermentation and optimization of process parameters for the production of PGL and PG by *Bacillus tequilensis* SV11-UV37

#### One-variable-at-a-time approach

The yield of the desired product under SSF depends on several factors like nature of the substrate, initial moisture content of the medium, the particle size of the substrate, incubation temperature, inoculum size and availability of nutrients etc.; hence, it is crucial to optimize the levels of these factors. For the initial optimization of process parameters, the one-variable-at-a-time approach was followed to enhance PGL and PG production by *B. tequilensis* SV11-UV37.

**Fig. 1:**
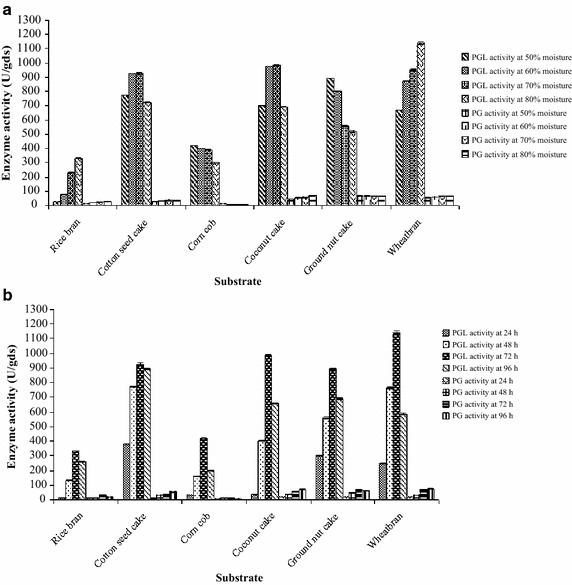
Effect of various factors on PGL and PG production: **a** effect of moisture level; **b** effect of incubation time using various agro-residues. *gds* gram dry substrate

To develop an efficient SSF process, suitable substrate selection is very important (Pandey 1992). An ideal solid substrate provides all the necessary nutrients required for the microorganism. Hence, in the present study various agro-residues were screened for the most suitable substrate for pectinolytic enzymes production. Among such, wheat bran was found to be the best substrate since it gave maximum PGL and PG yield using 80 % (v/w) moisture (Fig. 1a) at 72 h and 96 h (Fig. 1b) respectively; and then enzyme production declined. The reason could be the end product accumulation due to the nutrients scarcity that hampers enzyme production. Similarly, initial moisture content is one of the most critical parameters for the successful performance of SSF. The moisture content above the optimum level (80 %, v/w) showed a decline in enzyme production might be due to low porosity, low oxygen transfer, alteration in the wheat bran particle structure, gummy texture and poor adsorption of the enzyme to the substrate particles. Many reports have shown the optimum moisture level for pectinase production using SSF varies between 70 and 80 % for bacteria (Kashyap et al. 2003; Swain et al. 2009). The result is significant as wheat bran is inexpensive and prevalent (Kashyap et al. 2003), which makes the process more economical. The PG yield was highest at 96 h, which was similar to the results of Bayoumi et al. (2008). However, 72 h of incubation was chosen for further optimization studies since PGL production was much higher compared to that of PG.

**Fig. 2:**
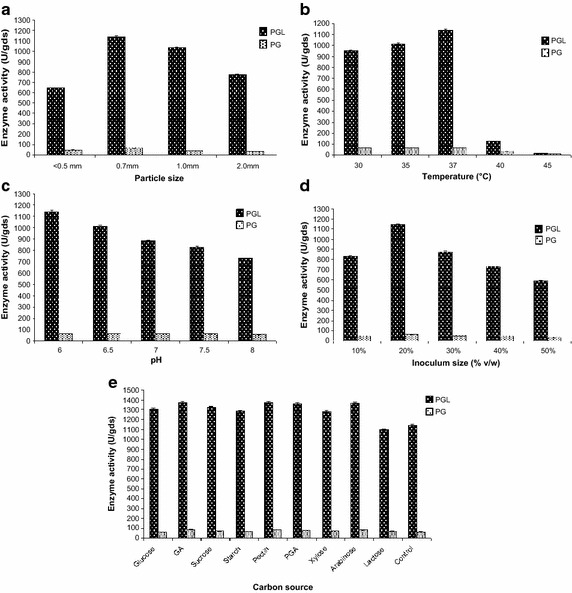
Effect of various factors on PGL and PG production: **a** effect of particle size of the wheat bran, **b** effect of temperature, **c** effect of pH, **d** effect of inoculum size and **e** effect of carbon source. *gds* gram dry substrate

The optimum particle size of wheat bran for the PGL and PG production was found to be 0.7 mm (Fig. 2a), which is in accordance with the results of Nicemol and Parukuttyamma (2008). The lower enzyme yield above and below the optimum level might be due to the reduction in total surface area and porosity of the medium.

Incubation temperature is an important factor which directly influences the microbial growth and metabolic activity of the enzymes. Also, it makes SSF process more significant due to the temperature increase of the fermenting mass during the fermentation because of respiration (Pandey and Radhakrishnan 1992). Similarly, the initial pH shows remarkable impact on membrane permeability of the microorganism, stability and biosynthesis of the enzyme (Murad and Salem 2001). In this study, the optimum temperature for PGL production was found to be 37 °C; however PG yield was slightly higher at 30 °C than 37 °C, which is negligible (Fig. 2b). The pH was observed to be optimum at 6.0 for PGL and PG production though there was no much difference in the PG yield between 6.0 and 7.0 (Fig. 2c). Hence, the present study showed optimum temperature and pH 37 °C and 6.0, respectively, could be due to the optimal growth of the microorganism and its maximum biomass production.

The age and size of the inoculum have a direct influence on the microbial growth and enzyme production. The current study also explains the same, where optimum inoculum size was identified as 20 % (v/w) for the PGL and PG production and lower enzyme yield was seen below and above the optimum level (Fig. 2d). The reason could be lower inoculum size might not support sufficient growth and biomass while higher inoculum may cause competition for nutrients. Sharma and Satyanarayana (2012) reported optimum enzyme titre with 25 % of a 24 h old inoculum.

Solid substrate supplies nutrients to the microorganisms besides working as an anchorage for the cells (Pandey et al. 2000). But, some of the nutrients are present in limited concentrations (Kashyap et al. 2003) hence external supplements are necessary to meet the required energy source for ample growth of the microorganism and its metabolism. Among the different ingredients supplied for the PGL and PG production; pectin, GA, arabinose and PGA induced the enzyme yield in the same order mentioned (Fig. 2e) while none of the nitrogen supplements and metal ions could enhance the enzyme production, which might be due to no further improvement in growth of the microorganism. Among the various carbon sources, pectin was detected as the most favored supplement for PGL (1371.25 U/gds) and PG (85.45 U/gds) production, and the similar results were reported by Islam et al. (2013) and Nicemol and Parukuttyamma (2008). The results of nitrogen supplementation concluded that external nitrogen additive is not necessary for PGL and PG production since the wheat bran has 65 % nitrogen (Martins et al. 2002), which itself provided the required nitrogen for the growth of the organism. Overall, among the several agro-residues screened for SSF, wheat bran was found to be an ideal substrate, and the maximum yield of pectate lyase (1371.25 U/gds) and polygalacturonase (85.45 U/gds) was obtained by using 80 % moisture, 0.7 mm particle size, 20 % inoculum and 1 % pectin at 37 °C, pH 6 in 3 days of incubation time.

#### Statistical approach

On the basis of results from one variable-at-a-time-approach, pectin, inoculum size and moisture and their levels were chosen for statistical optimization by RSM using CCD. Table 1 shows the experimental design together with the actual and predicted responses. The ANOVA showed that the model was highly significant with an F value of 112.64 for PGL (Table 2) and 30.27 for PG (Table 3) with a “p > F value” of <0.0001. In the case of PGL; the model terms B, C, AB, AC, BC, A^2^, B^2^ and C^2^, while A, B, C, AB, AC, BC and B^2^ for PG had a confidence level above 95 % (p < 0.05). This implied that the linear effect of inoculum size and moisture level, the interaction of each variable with the other and squared effects of all three variables for PGL; and linear and interaction effect of all three variables and squared effect of inoculum size for PG, were significant model terms. The coefficient of determination, R^2^ was found to be 0.9902 for PGL and 0.9646 for PG, indicates that this model can explain 99.02 and 96.46 % of the variability in the PGL and PG production, respectively. Predicted R^2^ of 0.9469 for PGL and 0.7771 for PG is in reasonable agreement with the adjusted R^2^ of 0.9814 and 0.9327. Adequate precision measures the signal to noise ratio and the model had a value of 38.182 for PGL and 22.427 for PG which indicated an adequate signal, generally a value greater than 4 is desirable. Further, a high similarity was observed between the predicted and experimental result.

**Table 2:** Analysis of variance: PGL

Source	Sum of squares	df	Mean square	F value	p value Prob > F	Significance
Model	1.340E+006	9	1.489E+005	112.64	<0.0001	Significant
A—Pectin (%)	17.52	1	17.52	0.013	0.9106	*
B—Inoculum size (%)	1.669E+005	1	1.669E+005	126.25	<0.0001	Significant
C—Moisture level (%)	2.026E+005	1	2.026E+005	153.27	<0.0001	Significant
AB	81582.75	1	81582.75	61.73	<0.0001	Significant
AC	47414.29	1	47414.29	35.88	0.0001	Significant
BC	4.915E+005	1	4.915E+005	371.88	<0.0001	Significant
A^2^	65595.72	1	65595.72	49.63	<0.0001	Significant
B^2^	40806.90	1	40806.90	30.88	0.0002	Significant
C^2^	2.088E+005	1	2.088E+005	157.97	<0.0001	Significant
Residual	13215.96	10	1321.60			Significant
Lack of fit	8615.47	5	1723.09	1.87	0.2539	*
Pure error	4600.50	5	920.10			
Cor total	1.353E+006	19				

* Means insignificant

**Table 3:** Analysis of variance: PG

Source	Sum of squares	df	Mean square	F value	p value Prob > F	Significance
Model	1202.23	9	133.58	30.27	<0.0001	Significant
A—Pectin (%)	243.93	1	243.93	55.27	<0.0001	Significant
B—Inoculum size (%)	62.81	1	62.81	14.23	0.0036	Significant
C—Moisture level (%)	425.03	1	425.03	96.30	<0.0001	Significant
AB	91.36	1	91.36	20.70	0.0011	Significant
AC	246.92	1	246.92	55.95	<0.0001	Significant
BC	46.06	1	46.06	10.43	0.0090	Significant
A^2^	0.14	1	0.14	0.031	0.8634	*
B^2^	72.96	1	72.96	16.53	0.0023	Significant
C^2^	7.62	1	7.62	1.73	0.2183	*
Residual	44.14	10	4.41			Significant
Lack of fit	31.50	5	6.30	2.49	0.1694	*
Pure error	12.63	5	2.53			
Cor total	1246.37	19				

* Means insignificant

Regression analysis of the data on enzyme yield was performed and the following second order polynomial equation was derived.

$$\begin{aligned} {\text{Y}}\; ( {\text{PGL)}} & = 1370.02 - 1.13{\text{A}} + 110.53{\text{B}} + 121.79{\text{C}} - 100.98{\text{AB}} \\ & \quad + 76.99{\text{AC}} - 247.86{\text{BC}} + 67.47{\text{A}}^{2} + 53.21{\text{B}}^{2} - 120.36{\text{C}}^{2} \\ {\text{Y}}\; ( {\text{PG)}} & = 90.12 - 4.23{\text{A}} - 2.14{\text{B}} + 5.58{\text{C}} - 3.38{\text{AB}} + 5.56{\text{AC}} \\ & \quad - 2.40{\text{BC}} - 0.098{\text{A}}^{2} - 2.25{\text{B}}^{2} + 0.73{\text{C}}^{2} \\ \end{aligned}$$

**Fig. 3:**
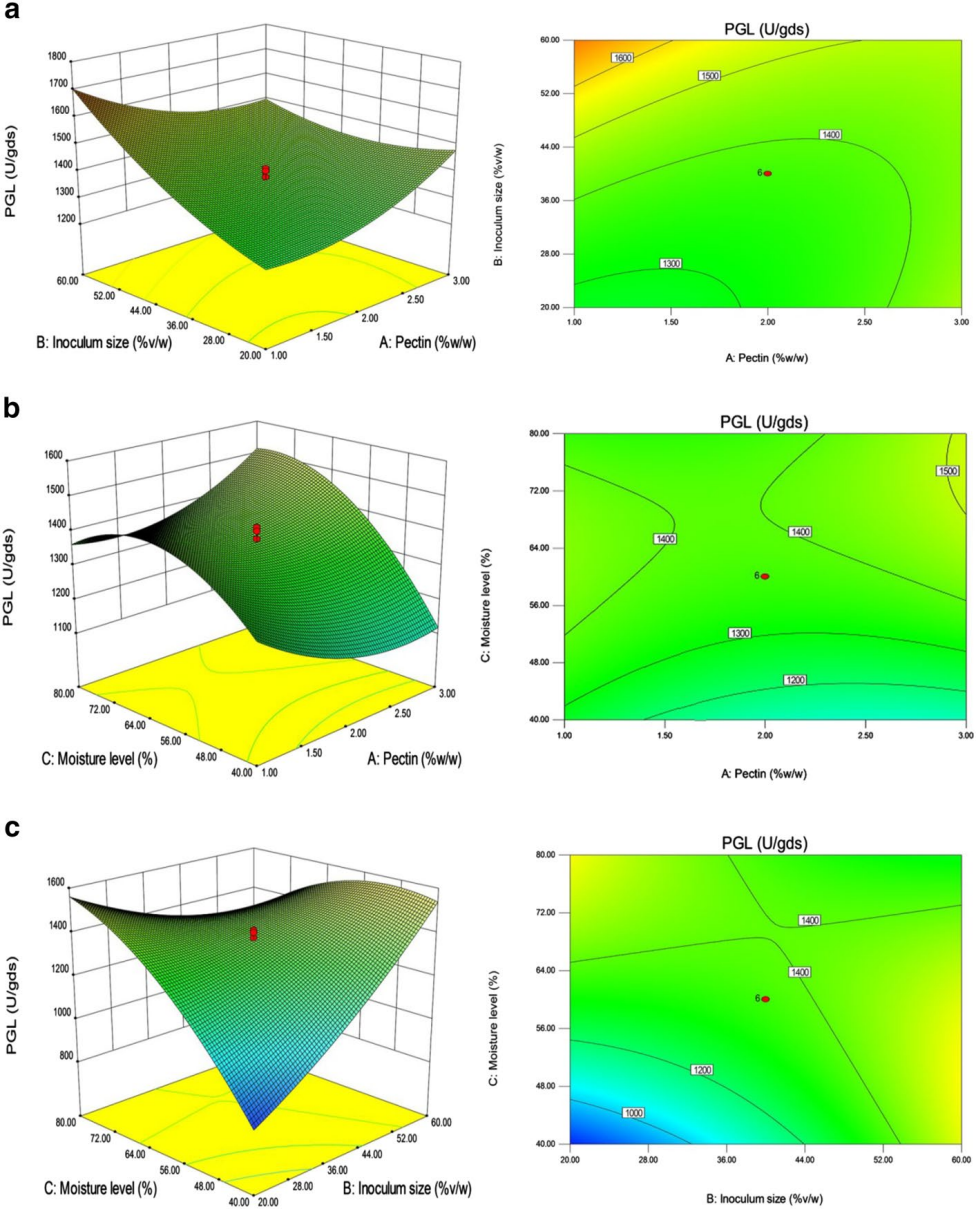
3D**-**response surface and contour plots: **a** interaction of pectin and inoculum size on PGL production, **b** interaction of pectin and moisture level on PGL production, **c** interaction of inoculum size and moisture level on PGL production. *gds* gram dry substrate

**Fig. 4:**
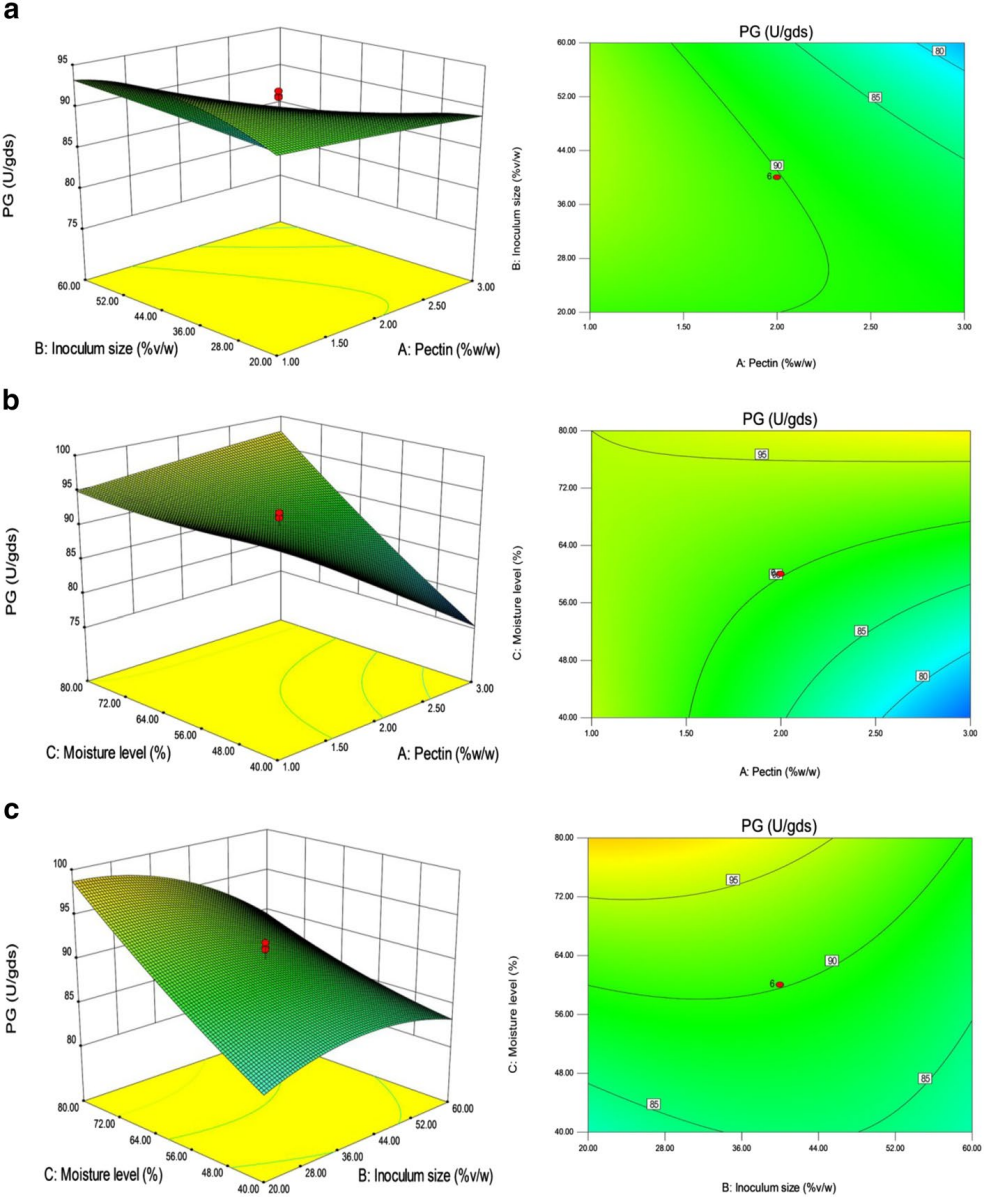
3D**-**response surface and contour plots: **a** interaction of pectin and inoculum size on PG production, **b** interaction of pectin and moisture level on PG production, **c** interaction of inoculum size and moisture level on PG production. *gds* gram dry substrate

Figures 3a and 4a depict 3D-response surface and contour plots of the interaction between pectin and inoculum size while keeping the moisture level at zero level. The result demonstrated that with the increase of pectin and inoculum size, PGL production gradually increased while PG production also slightly increased with the increase of inoculum size, but pectin did not show any obvious effect. The interaction of pectin and moisture level on PGL and PG production is illustrated in Figs. 3b and 4b, respectively. There was an increase in PGL yield with the increase of moisture level from 40 to 60 % when inoculum size fixed at 0 level, however, further increase of moisture level showed a gradual decline in the response. Further, the enzyme yield was slightly decreased with the increase of pectin concentration from 1 to 2 with no significant effect over 2 %. PG production was decreased with the increase of pectin concentration and there was no visible change in the yield with the increase of moisture level. Figures 3c and 4c show the interaction of inoculum size and moisture level on the yield of PGL and PG. It was observed that when pectin concentration fixed at 0 level, the increase of inoculum size and the moisture content resulted in the higher PGL yield. Although, PG production was increased with the increase of moisture level with insignificant effect at various inoculum levels.

##### Validation of the predicted model

The goal of optimization study was to find the optimal conditions which gave maximum enzyme yield. The software generated a solution to get the maximum response with the optimum conditions such as pectin-3 % w/w; inoculum size-20 % v/w and moisture level-80 % v/w. Validation of the model was performed with the suggested conditions and the results showed that the experimental values (1828.13 ± 9 U/gds PGL and 105.55 ± 2 U/gds PG) are close to the predicted values (1806.28 U/gds PGL and 103.33 U/gds PG), which indicated that the model was valid. On the whole, statistical optimization of selected parameters resulted in 1.6-fold increase in PGL and PG yield as compared to the yield obtained with the initial production medium. Similarly, Sharma and Satyanarayana (2012) also reported that the parametric optimization under SSF resulted in overall 1.7-fold improvement in enzyme production. Altogether, RSM was not only proved to be advantageous in identifying the optimum conditions for maximum PGL and PG production but also useful in evaluating the main and interaction effects of the process parameters over conventional method (one-variable-at-a-time approach).

### Application studies

Pectinolytic enzymes are often utilized in varied industrial sectors; specifically alkaline pectinases have a prime role in the retting and degumming as they remove interlamellar pectin present between the fibers. In the present study, retting of kenaf and sunn hemp, and degumming of sunn hemp were studied by employing PGL produced by *B. tequilensis* SV11-UV37. Although enzyme is a mixture of PGL and PG (about 18:1), here mentioned only PGL since PG yield is very less compared to PGL.

#### Retting of kenaf and sunn hemp fibers

In retting, producing a long fiber is a difficult task during the processing of bast plants. The standard strategies used for separating the long bast fibers are dew and water retting, each of those requires 14–28 days. Although the fibers made from water retting can be of prime quality, the long time and pollution have created this technique undesirable (Paridah et al. 2011). With the approach of biotechnology within the textile industry, new biological treatments emerge at recent times. Bacterial/enzymatic retting is superior to other retting processes by having much shorter retting time, higher fiber quality without significant damage and lower pollution (Hoondal et al. 2002; Van Sumere 1992).

**Fig. 5:**
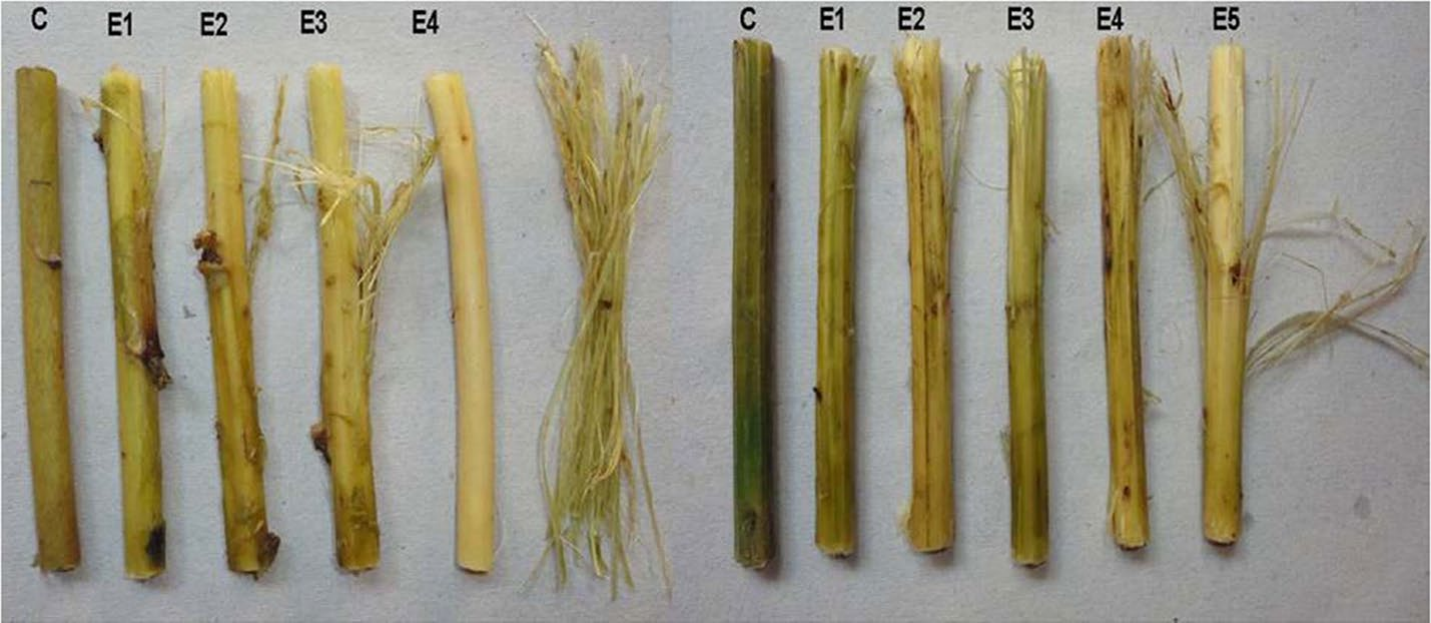
Effect of various enzyme dosages on retting of kenaf (*left*) and sunn hemp (*right*) fibers (*C* control, *E1* 100, *E2* 200, *E3* 300, *E4* 400 and *E5* 500 units of enzyme dose)

The results of retting experiments on kenaf (*H. cannabinus*) and sunn hemp (*Crotalaria juncea*) bast (stem) fibers representing the families of Malvaceae and Fabaceae, respectively, were shown in Fig. 5. As it is clearly seen from the Fried scores of the each natural fiber (Table 4), complete fiber separation was observed in kenaf in 24 h with 400 U, whereas partial separation (>75 %) was observed in sunn hemp even at 36 h, which might be due to its high tensile strength. Yadav et al. (2009) reported complete retting in *Cannabis sativa* and *Linum usitatissimum* while partial retting (Fried score of 3.5) in *C. juncea* fibers using purified pectin lyase (0.24 IU) at 37 °C in 24 h. However, there was a complete retting in *C. juncea* fibers when treated with pectin lyase purified from *Aspergillus flavus* (Yadav et al. 2008). In conclusion, the enzyme was found to be efficient in retting of kenaf and sunn hemp fibers, where it was able to separate fiber completely from kenaf and partly from sunn hemp.

**Table 4:** Retting of kenaf and sunn hemp fibers—fried score

Treatment time	Enzyme units	Kenaf	Sunn hemp
12 h	0	0	0
	100	1.3 (1,1,2)	0.3 (0,0,1)
	200	2.0 (2,2,2)	1.3 (1,1,2)
	300	3.3 (3,3,4)	2.0 (2,2,2)
	400	3.7 (3,4,4)	3.0 (3,3,3)
	500	4.3 (4,4,5)	3.3 (3,3,4)
24 h	100	2.3 (3,2,2)	1.0 (1,1,1)
	200	3.0 (3,3,3)	2.7 (3,3,2)
	300	4.3 (4,4,5)	3.7 (3,4,4)
	400	6.0 (6,6,6)	4.3 (5,4,4)
	500	6.0 (6,6,6)	5.0 (5,5,5)
36 h	100	2.7 (3,3,2)	1.7 (1,2,2)
	200	3.3 (3,3,4)	3.0 (3,3,3)
	300	5.0 (5,5,5)	4.3 (4,4,5)
	400	6.0 (6,6,6)	4.7 (5,5,4)
	500	6.0 (6,6,6)	5.0 (5,5,5)

#### Degumming of sunn hemp fibers

Generally, decorticated ramie and sunn hemp fibers possess up to 20–35 % of non-cellulosic gummy material comprising pectin and hemicellulose (Kochhar 1981; Bruhlmann et al. 1994). Consequently, it must be removed before their industrial usage. Sunn hemp fibers are mainly used in making of ropes, cords and twines etc., and they have high tensile strength, hence resistant to moisture, microorganisms. Compared to traditional degumming, which is achieved through a series of chemical treatments using hot alkaline solutions, microbial/enzymatic degumming is a more environmentally friendly method and consumes less energy. Microbial pectinases play an important role in the processing of these fibers as plant cambium cells have 40 % of the pectin in their dry weight (Bruhlmann et al. 1994; Bajpai 1999).

**Fig. 6:**
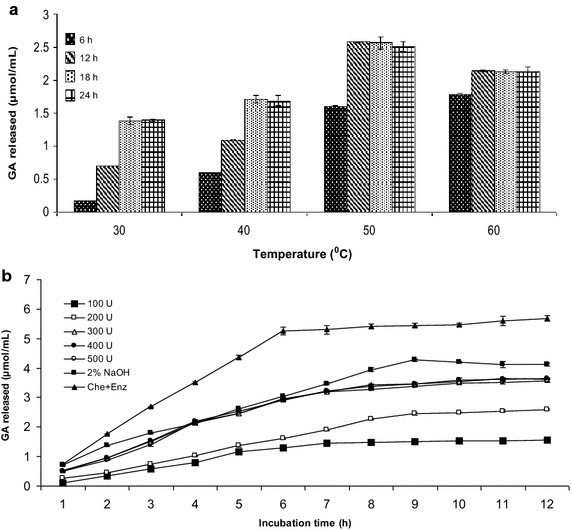
Degumming of sunn hemp fibers **a** effect of temperature, **b** effect of enzyme dose and reaction time on fiber treatment

**Fig. 7:**
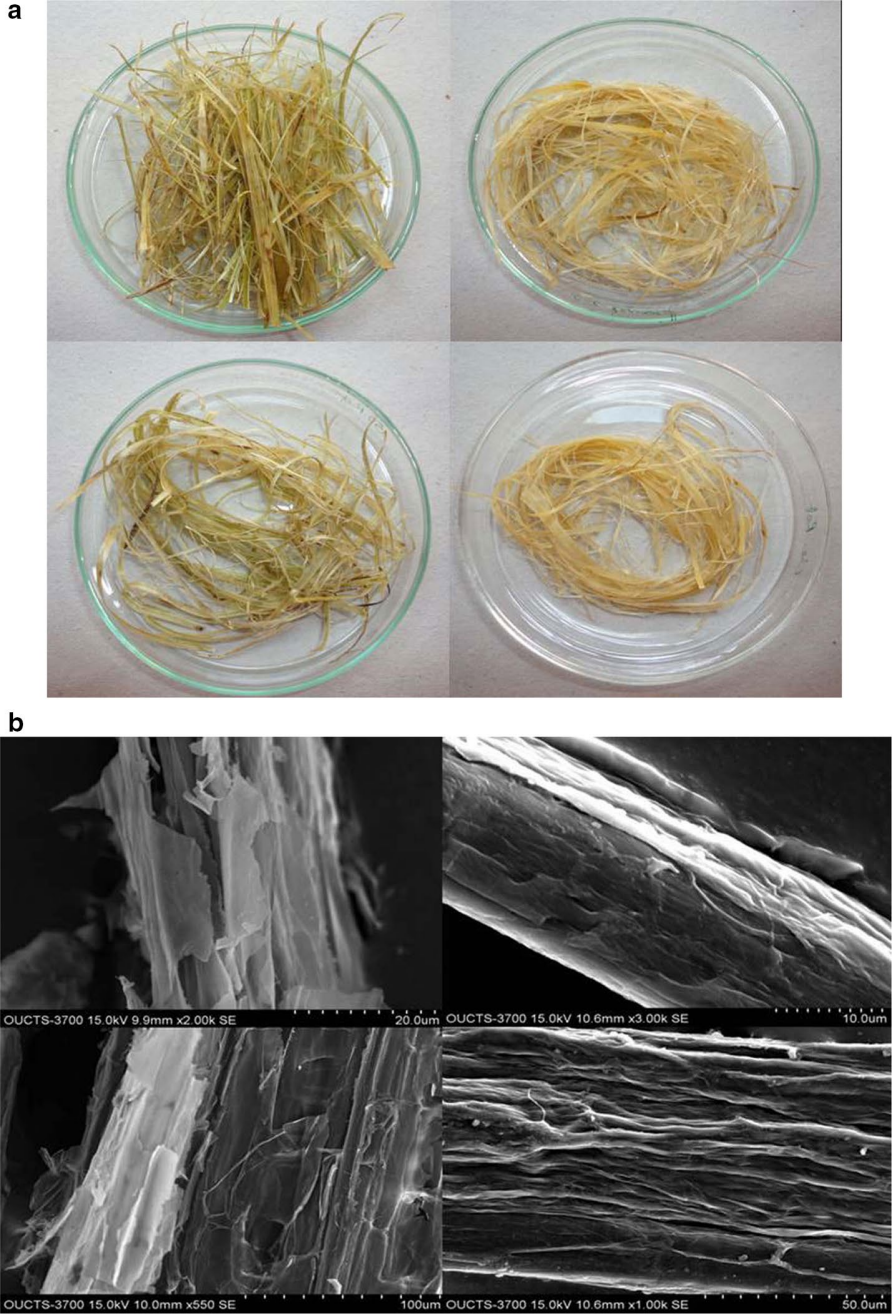
Degumming of sunn hemp fibers **a** texture and **b** scanning electron micrographs of sunn hemp fiber after different treatments (*upper left* untreated, *upper right* alkali treated, *lower left* enzyme treated, *lower right* alkali + enzyme treated)

In this study, the chemical degumming with NaOH, enzymatic degumming with crude alkaline pectate lyase and a combination of both processes were employed for the removal of gummy material from the decorticated sunn hemp fiber. The optimum temperature for the enzymatic degumming was found to be 50 °C (Fig. 6a) and the enzyme was stable for 24 h at this temperature with 75 % residual activity. Among the different enzyme doses used for degumming, the maximum GA (3.64 µmol/mL) was released with 300 U at 50 °C in 11 h (Fig. 6b). However, chemical degumming released more GA (4.27 µmol/mL) in less time (9 h) (Fig. 6b). When successive chemical and enzymatic treatment was performed, more GA (5.69 µmol/mL) was released in 12 h (Fig. 6b) compared to each individual treatment. The percentage weight loss of sunn hemp fiber after treatment with the enzyme, alkali and combination of both was 24.1, 43.35 and 54.5 %, respectively, which were in accordance to the GA released. The texture of the fiber after different treatments is shown in Fig. 7a. It is also clearly evident from the scanning electron microscopic pictures (Fig. 7b) that there was only partial removal of gummy material in both the chemical and enzymatic treatments while maximum removal was observed in combined treatment. Hence, from the results it has been concluded that chemical (alkali) treatment is required before the enzyme treatment, to make more prone to enzymatic action for the efficient removal of non-cellulosic gummy material (pectin) from the sunn hemp bast fibers. Similar results were reported by Kapoor et al. (2001), wherein 37 and 56 % reduction in the fiber weight with the release of 9.4 and 7.6 µmol/mL of reducing sugar was observed in the ramie and sunn hemp fibers respectively, by chemical and enzyme treatment. Kashyap et al. (2001) also studied the effectiveness of alkaline pectinase from *Bacillus* sp. DT7 in degumming of buel (*Grewia optiva*) bast fibers. In brief, the present degumming of sunn hemp study inferred that subsequent chemical and enzymatic treatment will effectively remove non-cellulosic gummy material from the fiber.

## Conclusions

Pectate lyase and polygalacturonase production from *B. tequilensis* SV11-UV37 is successfully enhanced by optimizing the process parameters with one-variable-at-a-time approach and RSM using wheat bran as a substrate. This makes an effective approach for pectin hydrolysis in a cost-effective and eco-friendly manner. Furthermore, application studies revealed that this enzyme mixture (major pectate lyase) may have possible usage in the fiber and textile industry by having good retting and degumming efficiency.
